# Deep-ultraviolet Raman scattering spectroscopy of monolayer WS_2_

**DOI:** 10.1038/s41598-018-29587-0

**Published:** 2018-07-30

**Authors:** Hsiang-Lin Liu, Teng Yang, Yuki Tatsumi, Ye Zhang, Baojuan Dong, Huaihong Guo, Zhidong Zhang, Yasuaki Kumamoto, Ming-Yang Li, Lain-Jong Li, Riichiro Saito, Satoshi Kawata

**Affiliations:** 1 Department of Physics, National Taiwan Normal University, Taipei, 11677 Taiwan; 2 Shenyang National Laboratory for Materials Science, Institute of Metal Research, Chinese Academy of Sciences, 72 Wenhua Road, Shenyang, 110016 China; 3 Department of Physics, Tohoku University, Sendai, 980-8578 Japan; 4 College of Sciences, Liaoning Shihua University, Fushun, 113001 China; 5 Department of Applied Physics, Osaka University 2-1 Yamadaoka, Suita, Osaka 565-0871 Japan; 6 Physical Science and Engineering Division, King Abdullah University of Science and Technology, Thuwal, 23955-6900 Saudi Arabia; 7 Research Center for Applied Science, Academia Sinica, Taipei, 10617 Taiwan; 8 Present Address: Department of Pathology and Cell Regulation, Graduate School of Medical Sciences, Kyoto Prefectural University of Medicine, 465 Kajii-cho, Kawaramachi-Hirokoji, Kamigyo-ku, Kyoto 602–8566 Japan

## Abstract

Raman scattering measurements of monolayer WS_2_ are reported as a function of the laser excitation energies from the near-infrared (1.58 eV) to the deep-ultraviolet (4.82 eV). In particular, we observed several strong Raman peaks in the range of 700∼850 cm^−1^ with the deep-ultraviolet laser lights (4.66 eV and 4.82 eV). Using the first-principles calculations, these peaks and other weak peaks were appropriately assigned by the double resonance Raman scattering spectra of phonons around the *M* and *K* points in the hexagonal Brillouin zone. The relative intensity of the first-order ${{\boldsymbol{E}}}_{{\bf{2}}{\boldsymbol{g}}}^{{\bf{1}}}$ to *A*_1*g*_ peak changes dramatically with the 1.58 eV and 2.33 eV laser excitations, while the comparable relative intensity was observed for other laser energies. The disappearance of the ${{\boldsymbol{E}}}_{{\bf{2}}{\boldsymbol{g}}}^{{\bf{1}}}$ peak with the 1.58 eV laser light comes from the fact that valley polarization of the laser light surpasses the ${{\boldsymbol{E}}}_{{\bf{2}}{\boldsymbol{g}}}^{{\bf{1}}}$ mode since the ${{\boldsymbol{E}}}_{{\bf{2}}{\boldsymbol{g}}}^{{\bf{1}}}$ mode is the helicity-exchange Raman mode. On the other hand, the disappearance of the *A*_1*g*_ peak with the 2.33 eV laser light might be due to the strain effect on the electron-phonon matrix element.

## Introduction

Layered transition metal dichalcogenides of hexagonal crystal structure (2H-TMDs) have attracted considerable attention in recent years. These materials exhibit distinct properties from their bulk counterparts because of reduced dimensionality and symmetry^1–5^, and offer unique opportunities for applications such as nanoelectronics, optoelectronics, spintronics, valleytronics, gas sensor, energy storage, and information processing^6–19^. Among 2H-TMDs, monolayer tungsten disulfide (WS_2_) is special in many respects. It has the largest direct band gap of about 2.1 eV at the *K* and $K^{\prime}$ points in the Brillouin zone^4,5,20^, resulting in the highest quantum efficiency of photoluminescence yield^4,5,21^. Furthermore, it exhibits sufficiently large exciton binding energy in the range of 0.3 ∼0.7 eV^20,22,23^, featuring stable A and B exciton absorptions even at room temperature. Additionally, it shows significant spin-orbit coupling that induces a large splitting of the valence band of about 0.4 eV^5,20,24^ at the *K* and $K^{\prime}$ points, leading to coupled spin and valley physics^25^. These superior properties make monolayer WS_2_ a very attractive material for use in field-effect transistors^26,27^, photodetectors^28–30^, solar cells^31^, light-emitting^21^, biosensing^32^, and spin valve devices^33^.

For many of these practical applications, knowledge of the lattice dynamics and electronic band structure of monolayer WS_2_ is important not only to characterize the structure but also to understand the optical and electronic properties of devices. Resonant Raman scattering spectroscopy has been proved to be an effective tool for probing such properties of monolayer WS_2_^34,35^, providing critical information about the phononic and electronic excitations in WS_2_ systems. In earlier studies, Berkdemir *et al*.^36^ examined the resonant Raman scattering spectra of monolayer WS_2_ with 488 nm (∼2.54 eV), 514.5 nm (∼2.41 eV), and 647 nm (∼1.92 eV) laser excitations. Gaur *et al*.^37^ studied the resonant enhancement of the first-order and second-order Raman phonon modes in monolayer WS_2_ with six different laser excitation wavelengths of 457.9 nm (∼2.71 eV), 476.5 nm (∼2.60 eV), 488 nm (∼2.54 eV), 496.5 nm (∼2.50 eV), 501.7 nm (∼2.47 eV), and 514.5 nm (∼2.41 eV). They both^36,37^ found that many second-order Raman phonon modes appear and an increase in the intensity of the longitudinal acoustic 2 *LA*(*M*) mode at 351 cm^−1^ occurs only when a 514.5 nm (∼2.41 eV) laser is resonant to the B exciton. This resonance can be explained in terms of the electron-phonon coupling based upon double resonant Raman scattering process. Corro *et al*.^38^ presented the results of Raman scattering spectra of monolayer WS_2_ using up to 25 laser excitation wavelengths in the visible range. They observed that the resonant excitation profiles of first-order *A*_1*g*_ and ${E}_{2g}^{1}$ and the second-order 2 *LA*(*M*) phonon modes show the intensity enhancements at 2.0, 2.4, and 2.7 eV, corresponding to three exciton absorption energies, revealing strong exciton-phonon interactions in monolayer WS_2_. Very recently, Yang *et al*.^39^ and Tan *et al*.^40^ investigated the excitation energy dependence of low-frequency Raman scattering spectra in few-layer WS_2_. Their results showed the quantum interference effects between low-frequency discrete phonon and exciton continuum under resonant excitation. Moreover, Miranda *et al*.^41^ explained the experimentally observed different resonant behavior of first-order ${A^{\prime} }_{1}$ and $E^{\prime}$ modes of monolayer MoTe_2_ in terms of the quantum interference between electronic transitions at differernt parts in the Brillouin zone.

Despite intense research having been conducted on resonant Raman scattering measurements of monolayer WS_2_ using the visible laser lines, their ultraviolet (especially for deep-ultraviolet) Raman scattering spectra have not been reported so far. Only our earlier study of the ultraviolet Raman scattering spectrum of monolayer MoS_2_ with the smaller band gap exhibits the rich second-order phonon structures^42^. Many high energy absorption peaks for monolayer WS_2_ were observed in the ultraviolet regime, showing much larger intensity than those of the A and B excitons^5,20^, which is due to the nesting effect at the Λ valley^43,44^ or Van Hove singularity at the *M* point. More recently, the stable, highly-responsive, and broadband (from 370 to 1064 nm) photodetection has been discovered in multilayer WS_2_^29^. These results show the great potential to use monolayer WS_2_ in the ultraviolet photodetector applications. Therefore, to gain further insight into the resonant Raman scattering spectra of monolayer WS_2_ using the ultraviolet laser lines is crucial for future design of effective ultraviolet photodetector based on this material. In this paper, we report a resonant Raman scattering study of monolayer WS_2_ with increasing laser excitation energies ranging from the near-infrared 785 nm (∼1.58 eV) to the deep-ultraviolet 257 nm (∼4.82 eV), and we compare our results with the predictions of first-principles calculations. We find that the anomalously strong enhancement of the Raman scattering spectra in the range of 700 ∼850 cm^−1^ as the second-order phonon modes by the deep-ultraviolet excitation wavelength 266 nm (∼4.66 eV) and 257 nm (∼4.82 eV). Furthermore, we observe the disappearance of ${E}_{2g}^{1}$ and *A*_1*g*_ peaks, respectively, with the 1.58 eV and 2.33 eV laser excitations. We discuss theoretically the origin of this disappearance.

The organization of the paper is as follows. In section II, we describe the technical details of the experiment and theoretical calculations. In section III, we present the experimental data and discuss the origin of the Raman scattering spectra by comparing with the results of first-principles calculations. Finally, the paper is summarized in Section IV.

## Technical Details

### Experiment

Monolayer WS_2_ thin films were grown on the sapphire substrates by chemical vapor deposition^45^. These thin films were single layer materials verified by atomic force microscopy^46^. Resonant micro-Raman scattering measurements were performed at room temperature using two deep-ultraviolet lasers at *λ* = 257 and 266 nm, a ultraviolet laser at *λ* = 354 nm^47,48^, two visible lasers at *λ* = 488 and 532 nm, and a near-infrared laser at *λ* = 785 nm. The power of all laser lines used was kept below 1.0 mW to avoid possible heating effects. The typical duration time of measuring the Raman scattering spectra was 300 seconds (*λ* = 257, 266, 354, and 785 nm) and 120 seconds (*λ* = 488 and 532 nm). A detailed description of the experimental Raman scattering setup is given elsewhere^42^. Spectroscopic ellipsometric measurements were performed for multiple angles of incidence between 60° and 75° by using a Woollam M-2000U ellipsometer over the spectral range from 0.73 to 6.42 eV. Optical absorption spectra were obtained through spectroscopic ellipsometry analysis using the stacked layer model (sapphire substrate/thin film/surface roughness/air ambient structure). The sample was placed in a continuous-flow helium cryostat for optical absorption measurement at 4.5 K.

### Theoretical model

We calculated the electronic band structure and phonon dispersion relation of monolayer WS_2_ based on first-principles density functional theory within the local density approximation (LDA) as implemented in the Quantum-Espresso code^49^. The monolayer WS_2_ separation from one unit cell to another unit cell was taken as 20 Å in the calculation to eliminate the inter-layer interaction. Projector augmented-wave (PAW) pseudopotentials^50,51^ was used with a plane-wave cutoff energy of 65 Ry to describe the interaction between electrons and ions. The electronic band structure with spin-orbit interaction considered was calculated using fully relativistic pseudopotentials derived from an atomic Dirac-like equation^52^. The atomic structure was fully relaxed with atomic force less than 10^−5^ Ry/Bohr. The Brillouin zone (BZ) was sampled over a *k*-mesh of 12 × 12 × 1 under the Monkhorst-Pack scheme^53^. The phonon energy dispersion relation of monolayer WS_2_ was calculated based on density functional perturbation theory^54^. The non-resonant Raman scattering intensity was calculated based on the Placzek approximation as introduced by Lazzeri and Mauri^55^. It is noted that in Quantum Espresso code, non-resonant Raman spectra based on the Placzek approximation can so far be taken care of only within local density approximation.

The optical absorption spectrum was calculated by the real (*ε*′) and imaginary ($\varepsilon ^{\prime\prime}$) parts of the dielectric function as a function of photon energy, respectively, based on the PAW methodology^56^ and the conventional Kramers-Kronig transformation. The absorption coefficient *α* is described by *α* = 4 *πκ E*_L_/(*hc*), where *E*_L_ is the incident laser excitation energy, *h* is the Planck constant, *c* is the speed of light, and *κ* is the extinction coefficient^57^, that is, $\kappa =\sqrt{(\sqrt{\varepsilon {^{\prime} }^{2}+\varepsilon {^{\prime\prime} }^{2}}-\varepsilon ^{\prime} )/2}$.

To evaluate the optical absorption as a function of laser energy *E*_L_ and wave vector in the BZ, the optical absorption probability^42^ was calculated as follows and normalized by $({{\rm{W}}}_{0}=\frac{2\pi {e}^{2}{\hslash }^{3}I}{{m}^{2}c{\varepsilon }_{0}})$,

1 $$W(\overrightarrow{k},{E}_{L})={W}_{0}\sum _{c,v}{|\frac{\overrightarrow{D}(c,v,\overrightarrow{k})\cdot \overrightarrow{P}}{{E}_{L}}|}^{2}\delta ({\varepsilon }_{c}(\overrightarrow{k})-{\varepsilon }_{v}(\overrightarrow{k})-{E}_{L}),$$

in which *m* is the electron mass, *I* is the intensity of the incident laser, *ε*_0_ is the dielectric constant for vacuum, $\overrightarrow{D}$(c, v, $\overrightarrow{k}$) (=$\langle {\psi }_{c}(\overrightarrow{k})|\nabla |{\psi }_{v}(\overrightarrow{k})\rangle$) is the dipole vector, and $\overrightarrow{P}$ is the laser polarization.

In order to evaluate the electron-phonon matrix element as a function of electron wavevector $\overrightarrow{k}$ in the first BZ for *q* = 0 phonon, we adopted the EPW package^58,59^ independently.

## Results and Discussion

**Figure 1:**
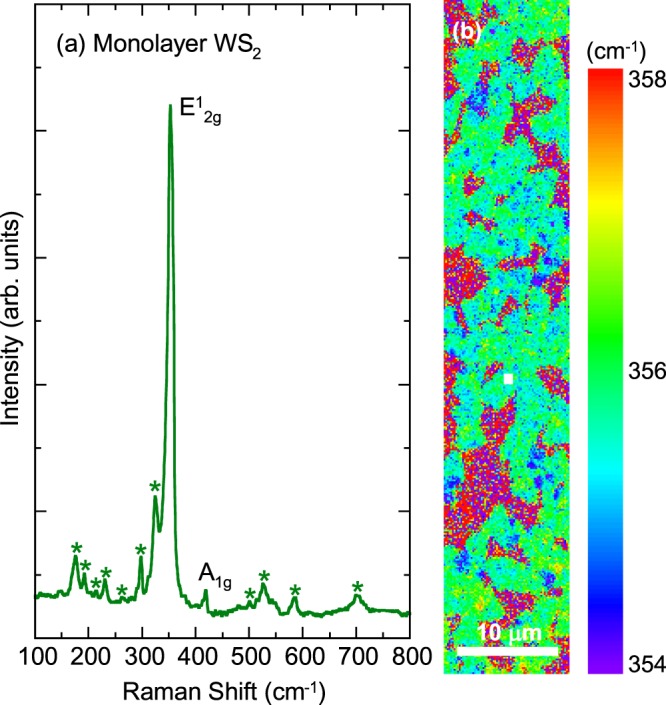
(**a**) The Raman scattering spectrum of monolayer WS_2_ excited by a 532 nm laser line. The asterisks denote the second-order Raman phonon modes. (**b**) The Raman mapping of the ${E}_{2g}^{1}$ peak frequency over the sample. The white dot in the middle of the image shows the place where the spectrum was collected in (**a**).

In Fig. 1(a), we show the Raman scattering spectrum of monolayer WS_2_ at room temperature excited by a 532 nm laser line. The spectrum exhibits two first-order Raman peaks with the labels of ${E}_{2g}^{1}$ and *A*_1*g*_ and several weak double resonant Raman structures as denoted by asterisks. We fitted these peaks by using a standard Lorentzian function. The two main peaks at approximately 356 and 417 cm^−1^ are associated with the zone center and first-order one-phonon emission for in-plane and out-of-plane vibrations with ${E}_{2g}^{1}$ and *A*_1*g*_ symmetries, respectively. The peak frequencies (356 cm^−1^ and 417 cm^−1^) well reproduce the previous Raman scattering measurements, indicating a single-layer signature^4,36–38^. Moreover, the spatial maps of the Raman frequency within 356 ± 2 cm^−1^ for the ${E}_{2g}^{1}$ mode (Fig. 1(b)) show uniform color contrast in each triangular WS_2_ domain. This evidence indicates that our monolayer WS_2_ is a high-quality sample. The assignment of the second-order Raman phonon modes will be discussed later.

**Figure 2:**
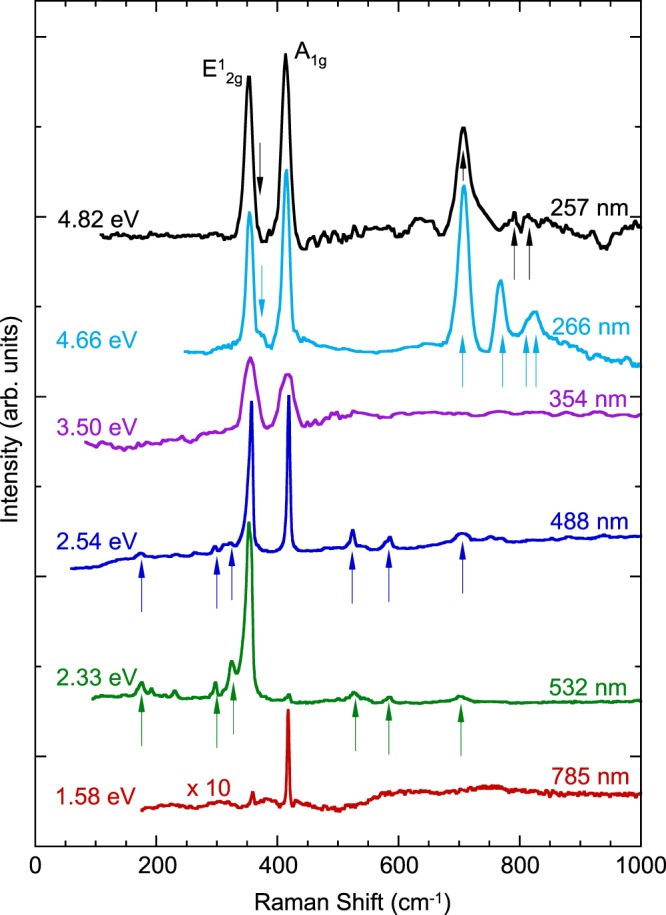
The Raman scattering spectra of monolayer WS_2_ excited by 785, 532, 488, 354, 266, and 257 nm laser lines. The arrows denote the double resonance peaks.

In order to further investigate the vibrational properties of monolayer WS_2_, we extended the Raman scattering measurements with excitation energies ranging from the near-infrared to deep-ultraviolet. In Fig. 2, we plot the Raman scattering spectra of monolayer WS_2_ excited by the near-infrared 785 nm (∼1.58 eV), visible 532 nm (∼2.33 eV) and 488 nm (∼2.54 eV), ultraviolet 354 nm (∼3.50 eV), and deep-ultraviolet 266 nm (∼4.66 eV) and 257 nm (∼4.82 eV) laser lights. There are three important features in the spectra. First, when the monolayer WS_2_ is excited at 785 nm, only *A*_1*g*_ and weak ${E}_{2g}^{1}$ Raman modes can be seen. The ${E}_{2g}^{1}$ mode is almost suppressed compared with the *A*_1*g*_ mode. By contrast, the opposite behavior is observed in the intensities of the ${E}_{2g}^{1}$ and *A*_1*g*_ modes for the 532 nm excitation. The possible origins of the disappearance of ${E}_{2g}^{1}$ or *A*_1*g*_ will be discussed later. Second, both ${E}_{2g}^{1}$ and *A*_1*g*_ modes show the prominent intensities excited by 488 nm, 354 nm, 266 nm, and 257 nm lasers. Additionally, many weak phonon modes appear in the Raman scattering spectra with 532 nm and 488 nm excitations as shown by arrows in Fig. 2. The peak positions of these weak modes shift to higher frequencies with increasing *E*_L_, suggesting that these modes are due to the second-order Raman scattering process^60–62^. Third, the intensities of these second-order phonon modes in the range of 700∼850 cm^−1^ become significant when the 266 nm and 257 nm lasers are applied.

**Figure 3:**
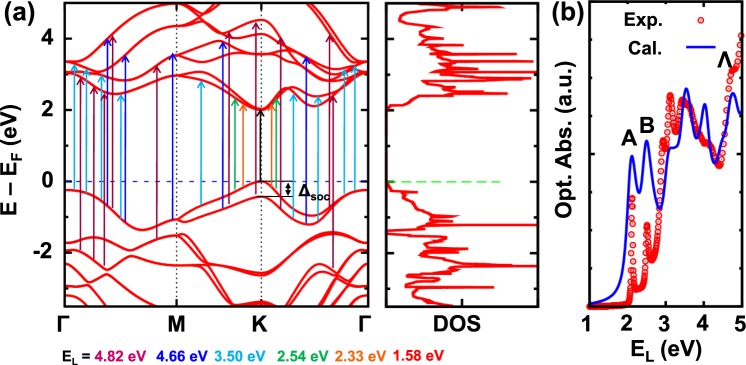
(**a**) Electronic band structure, density of states, and (**b**) optical absorption of monolayer WS_2_ measured at 4.5 K. The labels A, B, and Λ denote A, B, and Λ excitons.

To understand the origins of Raman scattering spectra due to different laser energies, we first calculate the electronic band structure and optical absorption. In Fig. 3(a), we show electronic band structure and density of states. The band splitting Δ_*soc*_ on top of valence band due to spin-orbit interaction is around 0.44 eV, which agrees very well with the energy splitting (∼0.42 eV) between A and B excitons in our experimental optical absorption data as indicated in Fig. 3(b) and also in the data by Rigosi *et al*.^63^. Electronic density of states (DOS) in Fig. 3(a) shows some typical features, such as constant value of DOS due to quadratic band dispersion at band edge of both valence and conduction bands around the zone-corner *K* point; and also Van-Hove singularity of DOS due to saddle points in the band structure around the zone-edge center *M* point. We expect a strong optical transition or Raman intensity for the optical transition for the transition energy of laser light at the Van-Hove singular DOS. Worth pointing out that due to an underestimate of optical band gap from density functional calculation, we upshift all conduction bands by 0.46 eV. With this band shift, our calculated optical absorption result in blue solid line in Fig. 3(b) agrees reasonably well with the experimental data (measured at 4.5 K) given in red empty dots, except for the absorption intensity that is calculated based on the single-particle picture without taking into account of electron-hole (exciton) interaction. Nevertheless, this difference is not relevant to the present analysis since we discuss the optical absorption for the transition energy with much larger energy than the exciton energies. It is noted that the A and B exciton peaks shift from 2.12 and 2.51 eV, respectively, at 4.5 K to 2.04 and 2.43 eV at 300 K. In Fig. 3(a), we mark the possible vertical optical transition by solid arrows for the laser lines used in the experiment. The electron excitation due to the laser lines of 532 nm (∼2.33 eV) and 488 nm (∼2.54 eV) takes place with wave vector close to the *K* point, suggesting that the Raman scattering spectra are due to the A or B excitons near the *K* point. In the case of *E*_L_ = 1.58 eV, since *E*_L_ is much smaller than the energy gap at the *K* point, the Raman scattering spectrum is non-resonant in which the dominant contribution of the Raman scattering intensity comes from the *K* point.

The vertical transition by the laser line in the ultraviolet 354 nm (∼3.50 eV) occurs more widely in the Brillouin zone, which gives a peak in the joint density of states at 3.50 eV. This situation explains the reason why the Raman peaks other than ${E}_{2g}^{1}$ and *A*_1*g*_ are almost invisible (the peaks become broad) compared with other laser lines because the double resonance wavevectors of phonon exists over the Brillouin zone. By contrast, the two laser lines 266 nm (∼4.66 eV) and 257 nm (∼4.82 eV) in the deep-ultraviolet region give rise to singular joint density of states (JDOS) at the Λ point (around $\frac{1}{2}\overrightarrow{{\rm{\Gamma }}{\rm{K}}}$), which leads to specified resonant electron-photon process for prominent double resonance Raman peaks^42,64^.

**Figure 4:**
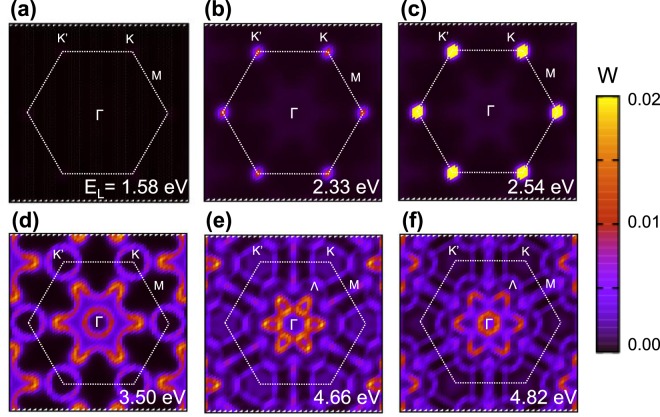
Optical absorption probability W of monolayer WS_2_ as a function of wavevector in the hexagonal Brillouin zone for the six different laser energies *E*_L_ = (**a**) 1.58 eV, (**b**) 2.33 eV, (**c**) 2.54 eV, (**d**) 3.50 eV, (**e**) 4.66 eV, and (**f**) 4.82 eV.

To assign the multiple resonant Raman peaks observed in Fig. 2, it is more straightforward to analyze the electron-photon resonant process in the whole Brillouin zone. In Fig. 4, we plot laser energy *E*_L_ dependence of optical absorption probability W of monolayer WS_2_ in the BZ. Consistent with the previous analysis in the band structure in Fig. 3(a), the wave vector *k*_*eγ*_ of electron-photon resonance process for the laser excitation energies of 2.33 and 2.54 eV is around *K* point, as shown in Fig. 4(b) and (c) while there is no resonant optical absorption in Fig. 4(a). Therefore the Γ-point or *K*-point phonons are expected to contribute to the intra-band or inter-band resonant Raman peaks, respectively. However, the *k*_*eγ*_ for the ultraviolet and deep-ultraviolet lasers is more complicated. Nevertheless, *k*_*eγ*_ for the ultraviolet and deep-ultraviolet lasers is along $\overrightarrow{{\rm{\Gamma }}K}$ line, as seen from Fig. 4(d–f).

**Figure 5:**
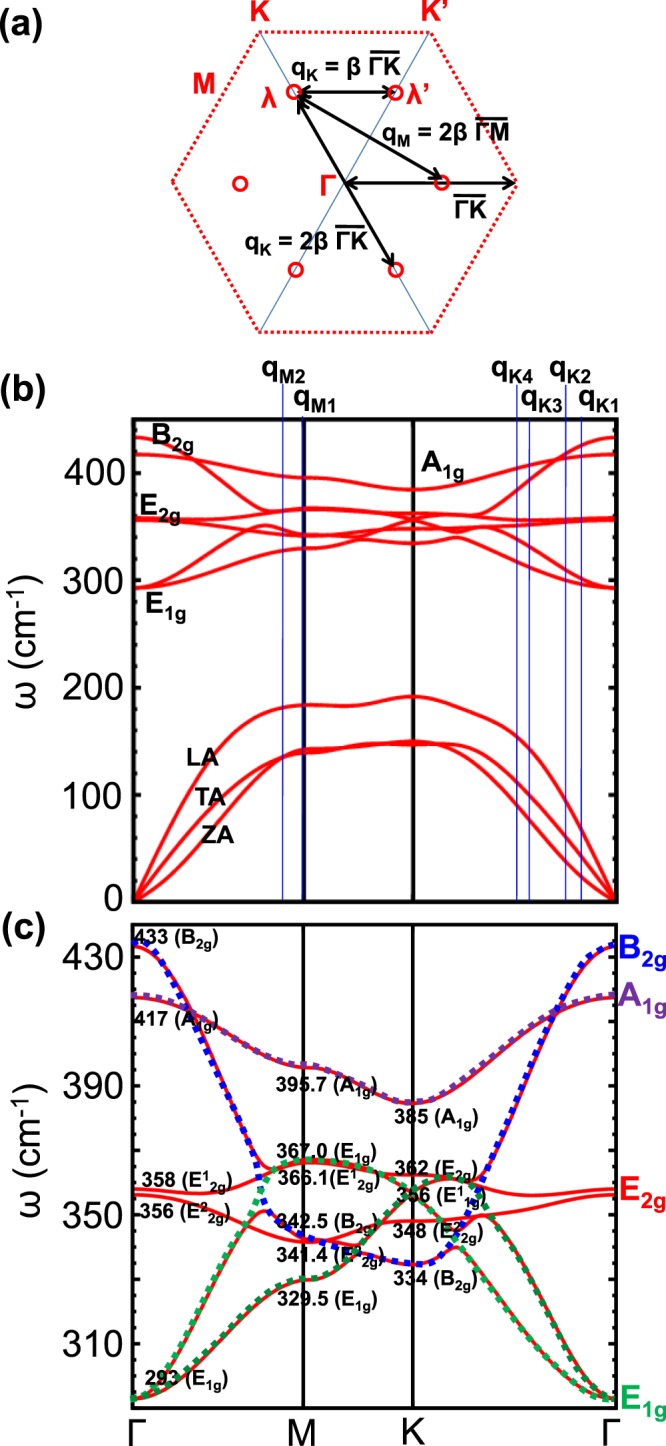
(**a**) The intervalley wave vector q _*e*−*ph*_ of resonance electron-phonon scattering process between *λ* and $\lambda ^{\prime}$ where electron-photon resonance process occurs. *λ* point is defined as a *k* point along $\overline{{\rm{\Gamma }}M}$ line and becomes Λ point at 1/2 $\overline{{\rm{\Gamma }}M}$ line. (**b**) Phonon dispersion relation of monolayer WS_2_. The blue vertical lines denote the wave vectors of double resonance Raman scattering. (**c**) Optical phonon dispersion relation. Dashed lines in color are used to highlight the dispersion of different optical phonon bands.

To extract the phonon wave vector *q* of resonance electron-phonon scattering process for the ultraviolet and deep-ultraviolet lasers, we analyze the possible intervalley/intravalley scattering between *λ* or $\lambda ^{\prime}$ points as shown in Fig. 5(a). *λ* point is defined as a *k* point along $\overline{{\rm{\Gamma }}K}$ line and becomes Λ point at 1/2 $\overline{{\rm{\Gamma }}K}$ line. Starting at the *λ* points with *k*_*eγ*_ = *β* $\overline{{\rm{\Gamma }}K}$, the phonon wavevector *q* can be either *q*_*K*_ = *β* $\overline{{\rm{\Gamma }}K}$, or *q*_*M*_ = 2 *β* $\overline{{\rm{\Gamma }}M}$, or *q*_*K*_ = 2 *β* $\overline{{\rm{\Gamma }}K}$. Here the value of *β* is a function of *E*_L_, as seen from Fig. 4, for example, *β* takes two values (*β* = 0.21 and 0.43) for *E*_*L*_ = 4.66 eV, and another two values (*β* = 0.17 and 0.50) for *E*_*L*_ = 4.82 eV. The corresponding phonon wave vectors *q* which satisfied the double resonant condition, including *q*_*M*_ near the *M* point and *q*_*K*_ close to the *K* point, are marked in blue lines in the phonon dispersion relation as shown in Fig. 5(b). We have pointed out in the previous work^42,65^ that Van Hove singularity of both electronic and phonon density of states at the *M* point can give rise to the resonant electron-photon and electron-phonon process, which can significantly enhance the Raman scattering intensity. The Raman peaks above 700 cm^−1^ with large intensity due to both 266 nm and 257 nm laser lines, as seen from Fig. 2, are assigned to the combination mode or overtone mode at the *M* point (*q*_*M*1_) or close to the *M* point (*q*_*M*2_ = 0.86 $\overline{{\rm{\Gamma }}M}$), as indicated in Fig. 5(b) and summarized in Table 1.

**Table 1:** Phonon frequencies (cm^−1^) in the second-order Raman scattering spectra of monolayer WS_2_ excited by the 2.33, 2.54, 4.66, and 4.82 eV laser lines. A comparison is given between the experimentally unassigned modes and the calculated second-order phonon spectra at high-symmetry *M*, *K*, and Γ points.

*E*_*L*_(eV)	*ω*_*exp*_(cm^−1^)					
2.33	176	298	324	526	586	704
2.54	176	298	322	524	586	706
	*ω*_*theory*_(cm^−1^)					
2.33,2.54	171	296	334	531	584	710
mode	${{\rm{E}}}_{2g}^{1}$(K) − LA(K)	2TA(K)	${{\rm{B}}}_{2g}^{1}$(K)	A_1*g*1*g*_(K) + ZA(K)	2E _1*g*_(Γ)	${{\rm{E}}}_{2g}^{1}$(K) + ${{\rm{E}}}_{2g}^{2}$(K)
*E*_*L*_(eV)	*ω*_*exp*_(cm^−1^)					
4.66	369	708	768	824	816	
4.82	369	708	743	792	826	
	*ω*_*theory*_(cm^−1^)					
4.66	367	707	763	821	814	
4.82	367	707	737	791	823	
mode	2LA(M)	${{\rm{E}}}_{2g}^{1}$(M) + ${{\rm{E}}}_{2g}^{2}$(M)	A_1*g*_(M) + ${{\rm{E}}}_{2g}^{2}$(M)	2A _1*g*_(M)	2A _1*g*_(K)	

Since all these resonant Raman peaks for the ultraviolet and deep-ultraviolet lasers are due to optical phonon modes, we show the optical phonon dispersion relation as shown in Fig. 5(c). Compared to the well dispersive *B*_2*g*_ (dashed blue line) and *E*_1*g*_ modes (dashed green lines) along the high-symmetry line, *A*_1*g*_ (dashed purple line) and *E*_2*g*_ (red solid lines) modes are relatively flat and believed to contribute to the small shift of those resonant Raman peaks around 700 cm^−1^ to 708 cm^−1^ due to both 266 nm and 257 nm laser lines. In particular, the ${E}_{2g}^{1}$ (M) and ${E}_{2g}^{2}$(M) modes have an opposite energy dispersion to each other, the combinational resonant Raman peak from the two modes should have no obvious laser-energy dependence, such as the pronounced Raman peak at 708 cm^−1^ from 266 nm to 257 nm laser line.

In Table 1, we list up the observed weak Raman scattering spectra excited by 2.33, 2.54, 4.66, and 4.82 eV laser lines and the assignment to the double resonance Raman scattering spectra. The upper part of the Table 1 gives the assignment of the Raman peaks excited by the visible light (2.33 and 2.54 eV). Since all the assigned combination, difference combination and overtone modes are due to the *K*-point phonons, no laser energy dependence of Raman frequency is expected. The lower part of the Table 1 shows the assignment of Raman peaks due to the deep-ultraviolet lasers (4.66 and 4.82 eV). As discussed above, phonon at or near the *M* point are responsible for the pronounced Raman peaks. Except for the 2 *LA*(*M*) and ${E}_{2g}^{1}$ + ${E}_{2g}^{2}$ modes, an obvious laser energy dependence of Raman peaks due to the deep-ultraviolet lasers is observed both in the experiment and theory, which is an evidence that the assignment of double resonance Raman peak is consistent with phonon dispersion relation.

Let us briefly discuss the disappearance of *A*_1*g*_ intensity at *E*_L_ = 2.33 eV and ${E}_{2g}^{1}$ intensity at *E*_L_ = 1.58 eV. In order to check the reproducibility of the relative intensity, we measured the Raman scattering spectra at three different spots. The relative intensity at *E*_L_ = 2.33 eV is almost identical to that measured by Corro *et al*.^38^ at 530.9 nm laser line. Corro *et al*.^38^ attributed this to the exciton-phonon interaction between the B exciton and *A*_1*g*_ phonon. However, Carvalho *et al*.^66^ observed in MoS_2_ and MoSe_2_ an enhancement of *A*_1*g*_ peak at the energy of the B exciton and explained that the ${d}_{{z}^{2}}$ orbital can couple with the *A*_1*g*_ mode other than with the ${E}_{2g}^{1}$ mode. Following the analysis by Carvalho *et al*.^66^, we calculated the wavefunctions of the 5 ${d}_{{z}^{2}}$ orbital of W and the 4 ${d}_{{z}^{2}}$ orbital of Mo, we found that the delocalization of atomic orbitals is similar to each other. In fact, the lattice constants of WS_2_ (*c* = 3.19 Å) and MoS_2_ (*c* = 3.19 Å) are almost identical. But the result for WS_2_ is opposite to that of MoS_2_ and MoSe_2_. The previous exciton-phonon effect between the *A*_1*g*_ mode and the B exciton can not apply to WS_2_. It is pointed out that the disappearance of the *A*_1*g*_ mode at the energy of the C exciton (MoS_2_ at *E*_L_ = 2.75 eV, MoSe_2_ at *E*_L_ = 2.60 eV) can be explained by the exciton-phonon interaction according to the discussion by Carvalho *et al*.^66^. Though we do not have the Raman scattering spectra of WS_2_ at the C exciton energy (2.80 eV), Corro *et al*. showed the disappearance of *A*_1*g*_ mode at 457.9 nm and 472.7 nm laser lines^38^.

**Figure 6:**
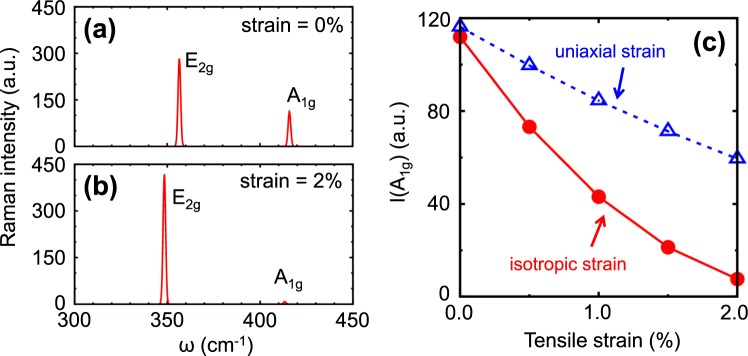
Non-resonant Raman scattering spectrum calculated based on the Placzek polarizability theory. Raman scattering spectuma at (**a**) zero and (**b**) 2% isotropic tensile strain. (**c**) Raman scattering intensity of *A*_1*g*_ mode as a function of strain for both uniaxial and isotropic strains.

Here we try to consider two possible origins of the disappearance of the *A*_1*g*_ mode. One of possible origins is due to the node of electron-phonon matrix element around the *K* point^64^. Since we do not calculate directly the exciton-phonon matrix element that is given by weighted sum of electron-phonon matrix element^67^, we can not specify the energy in which the exciton-phonon matrix element becomes zero. It should be mentioned that the laser energy that gives zero electron-phonon matrix element is 3.06 eV that is much larger than 2.33 eV even if we consider the exciton binding energy. Another possible reason for the disappearance of the *A*_1*g*_ mode is the strain effect of the Raman scattering intensity. In Fig. 6(a,b), we show the non-resonant Raman scattering spectra calculated based on the Placzek polarizability theory^55^ at both (a) zero and (b) 2% isotropic tensile strain. In Fig. 6(c), we show the strain dependence of *A*_1*g*_ intensity as a function of strain and *A*_1*g*_ intensity exponentially decreases with increasing isotropic tensile strain. It is noted that the *A*_1*g*_ intensity does not decrease much for uniaxial strain. We expect that the *A*_1*g*_ disappearance at 2.33 eV may have something to do with lattice tensile strain effect possibly due to laser heating. However, since we did not study power dependence of Raman scattering spectra, we could not see if the strain effect is essential of not. And 2% strain is relatively large from the thermal expansion or the interlayer interaction between WS_2_ and the sapphire substrate.

As for the disappearance of ${E}_{2g}^{1}$ at *E*_L_ = 1.58 eV, since this Raman scattering process is non-resonance, the main contribution to Raman scattering intensity is the *K*-point optical absorption which is valley-polarized. That means that only left-handed (or right-handed) component of the circular polarized light is absorbed and emitted at the *K*(or $K^{\prime}$) point. Since ${E}_{2g}^{1}$ mode changes the helicity of circular polarized light in the scattered light, the Raman scattering process of ${E}_{2g}^{1}$ is suppressed by valley polarization. It is the reason why ${E}_{2g}^{1}$ is suppressed for 1.58 eV. It is important to note that this effect of valley polarization occurs even when incident light is linearly polarized. The linear polarized light is expressed by the sum of left-handed and right-handed circular light for each of which the optical absorption occurs at the *K* and $K^{\prime}$ points.

## Summary

In summary, we report a combined experimental and theoretical study of the deep-ultraviolet Raman scattering spectra of monolayer WS_2_ in which we observed new intense Raman peaks in the range of 700∼850 cm^−1^, which can be assigned to the double resonance Raman scattering spectra with the phonon wave vector connecting the Λ points. Though the peaks show dispersive behavior of Raman frequency with increasing *E*_*L*_, the other Raman peaks show non-dispersive nature because of the opposite phonon dispersion to each other for a combination modes of the ${E}_{2g}^{1}$ and ${E}_{2g}^{2}$ modes. The disappearance of *A*_1*g*_ peak with the 2.33 eV laser excitation is probably from a lattice tensile strain due to laser heating, while disappearance of ${E}_{2g}^{1}$ peak with the 1.58 eV laser excitation is due to valley polarization effect and helicity exchanged Raman process of the ${E}_{2g}^{1}$ mode.
